# Differences in swallowing and general caregiver burden between child and spouse caregivers of people living with dementia

**DOI:** 10.1002/alz70858_104426

**Published:** 2025-12-26

**Authors:** Anittha Mappanasingam, Harmonie Chan, Jyothy Nair, Samantha Shune, Malene Stewart, Nan Uzbalis, Ashwini Namasivayam‐MacDonald

**Affiliations:** ^1^ McMaster University, Hamilton, ON, Canada; ^2^ University of Oregon, Eugene, OR, USA

## Abstract

**Background:**

Family caregivers of people living with dementia (PwD) experience high levels of burden, which has been reported to increase in the presence of dysphagia (swallowing difficulties). As dysphagia is common among PwD, addressing caregiving challenges is critical to prevent increased burden. Research suggests differences in general caregiving experiences between spouses and child caregivers, where child caregivers report more emotional and financial burden than spouses who report more social burden. However, no research exists on the differences in swallowing‐specific caregiver burden between these groups. Thus, the aim of this study was to explore differences in general and swallowing‐related caregiver burden between child and spouse caregivers of PwD.

**Method:**

The study included unpaid, English‐speaking child and spouse caregivers of a family member with dementia or cognitive impairment residing in Canada. The Zarit Burden Interview (ZBI; score range: 0 to 88) assessed general caregiver burden. The Caregiver Analysis of Reported Experiences with Swallowing Disorders (CARES) screening tool (score range: 0 to 26) assessed swallowing‐related caregiver burden. An independent sample t‐test and Mann‐Whitney U test assessed differences in general and swallowing‐related caregiver burden, respectively, between child and spouse caregivers. For each CARES item, binomial logistic regression was performed to assess whether responses differed across spouse and child caregivers.

**Result:**

Fifty‐three spouse caregivers (mean±SD age=70±12) and 120 child caregivers (mean±SD age=51±13) participated in the study. Mean±SD ZBI score was 38±17 for spouses and 37±18 for child caregivers. Median(IQR) CARES score was 9(18) for spouses and 9(14) for child caregivers. There was no significant difference between spouse and child caregivers when assessing general caregiver burden (t(171)=0.29, *p* >0.05) or swallowing‐related caregiver burden scores (z=‐0.04, *p* >0.05). Spouses were more likely than child caregivers to report increased mealtime‐ and nutrition‐related responsibilities (OR=2.96, 95%CI=1.11,7.92), difficulty in making frequent plans (OR=2.54, 95%CI=1.06,6.10), and lack of support from care recipients (OR=6, 95%CI=2.24,14.67).

**Conclusion:**

Findings suggest that swallowing‐related caregiving experience of PwD may differ between spouses and child caregivers. Identifying these differences allows for the development of unique resources for each group based on their specific concerns. Future research with larger samples is needed to further explore these differences.

